# Midlife type 2 diabetes and poor glycaemic control as risk factors for cognitive decline in early old age: a post-hoc analysis of the Whitehall II cohort study

**DOI:** 10.1016/S2213-8587(13)70192-X

**Published:** 2014-03-06

**Authors:** Richard H Tuligenga, Aline Dugravot, Adam G Tabák, Alexis Elbaz, Eric J Brunner, Mika Kivimäki, Archana Singh-Manoux

**Affiliations:** a INSERM U1018, Centre for Research in Epidemiology and Population Health, Hôpital Paul Brousse, Villejuif, France; b Université Paris Sud 11, Paris, France; c Departments of Epidemiology and Public Health, University College London, London, UK; d Centre de Gérontologie, Hôpital Ste Périne, AP-HP, Paris, France; e Université de Versailles St-Quentin-en-Yvelines, Versailles, France; f First Department of Medicine, Semmelweis University Faculty of Medicine, Budapest, Hungary

## Abstract

**Background:**

Type 2 diabetes increases the risk for dementia, but whether it affects cognition before old age is unclear. We investigated whether duration of diabetes in late midlife and poor glycaemic control were associated with accelerated cognitive decline.

**Methods:**

5653 participants from the Whitehall II cohort study (median age 54·4 years [IQR 50·3–60·3] at first cognitive assessment), were classified into four groups: normoglycaemia, prediabetes, newly diagnosed diabetes, and known diabetes. Tests of memory, reasoning, phonemic and semantic fluency, and a global score that combined all cognitive tests, were assessed three times over 10 years (1997–99, 2002–04, and 2007–09). Mean HbA_1c_ was used to assess glycaemic control during follow-up. Analyses were adjusted for sociodemographic characteristics, health-related behaviours, and chronic diseases.

**Findings:**

Compared with normoglycaemic participants, those with known diabetes had a 45% faster decline in memory (10 year difference in decline −0·13 SD, 95% CI −0·26 to −0·00; p=0·046), a 29% faster decline in reasoning (−0·10 SD, −0·19 to −0·01; p=0·026), and a 24% faster decline in the global cognitive score (−0·11 SD, −0·21 to −0·02; p=0·014). Participants with prediabetes or newly diagnosed diabetes had similar rates of decline to those with normoglycaemia. Poorer glycaemic control in participants with known diabetes was associated with a significantly faster decline in memory (−0·12 [–0·22 to −0·01]; p=0·034) and a decline in reasoning that approached significance (−0·07 [–0·15 to 0·00]; p=0·052).

**Interpretation:**

The risk of accelerated cognitive decline in middle-aged patients with type 2 diabetes is dependent on both disease duration and glycaemic control.

**Funding:**

US National Institutes of Health, UK Medical Research Council.

## Introduction

Dementia represents a serious public health challenge because of ageing populations worldwide.^1^ The prevalence of type 2 diabetes is also rising rapidly around the world, and increases the risk of dementia, including Alzheimer's disease.^2^, ^3^, ^4^ Several studies have shown poorer cognitive performance and faster cognitive decline in people with diabetes than in those without the disease.^5^, ^6^, ^7^, ^8^, ^9^ However, with some notable exceptions,^5^, ^7^, ^9^ most of the evidence for the effect of type 2 diabetes on cognitive ageing comes from studies in elderly populations.^6^, ^8^ Typically, such research is based on adults aged 65 years or older at the start of the study, with follow-up measurement of incident dementia. Some researchers believe that diabetes does not necessarily affect cognition before old age,^10^, ^11^ the implication being that the association between the two exists not because diabetes is a risk factor for dementia, but because of shared risk factors such as hypertension. Since dementia is a progressive disease involving cognitive decline over several years,^12^, ^13^ investigation is needed to determine whether diabetes affects cognitive decline before old age.

Our aim was to assess whether, compared with normoglycaemia, type 2 diabetes and prediabetes^14^ are associated with faster cognitive decline from late midlife (age 55 years) to early old age (age 65 years), an age range in which dementia is uncommon. We also aimed to examine the possibility of a dose-response relation by investigating the role of duration of diabetes (the underlying hypothesis being that if diabetes is a risk factor for cognition then longer exposure to diabetes would have a stronger effect on cognitive decline) and to investigate whether glycaemic control (measured by HbA_1c_), including among individuals with type 2 diabetes, is associated with cognitive decline.

## Methods

### Study design and participants

The Whitehall II study was established in 1985 with the recruitment of British civil servants aged 35–55 years from 20 London-based departments to investigate determinants of chronic diseases, with the baseline assessment taking place in 1985–88.^15^ Clinical examinations were also done in 1991–93, 1997–99, 2002–04, and 2007–09. Cognitive testing was introduced to the study in 1997–99 and repeated in 2002–04 and 2007–09. Written informed consent from participants and research ethics approvals (University College London ethics committee) were renewed at each contact; the most recent approval was from the Joint University College London/University College London Hospital Committee on the Ethics of Human Research (Committee Alpha), reference number 85/0938.

### Measurements

We ascertained type 2 diabetes status in 1991–93 and 1997–99. We took venous blood samples after a minimum 5 h fast, followed by a 75 g, 2 h oral glucose tolerance test. Blood samples were drawn into fluoride monovette tubes and centrifuged on site. We measured blood glucose using the glucose oxidase method.^16^ Type 2 diabetes was defined by WHO criteria,^17^ based on a fasting glucose of 7·0 mmol/L or more, or a 2 h postload glucose of 11·1 mmol/L or more. Participants who met these criteria at the 1991–93 examination or who had known diabetes in 1997–99 (ie, doctor-diagnosed diabetes or use of antidiabetic drugs) were classified as known diabetes. Those without a history of diabetes, but who met the diabetes criteria at the 1997–99 examination were classified as newly diagnosed diabetes. Non-diabetic participants were classified as prediabetic if their fasting plasma glucose concentration was between 6·1 and less than 7·0 mmol/L and their 2 h postload glucose concentration was less than 7·8 mmol/L (impaired fasting glucose), or if their fasting glucose was less than 7·0 mmol/L and their 2 h postload plasma glucose concentration was between 7·8 and less than 11·1 mmol/L (impaired glucose tolerance).^17^ Others were classified as normoglycaemic.

Glycaemic control was characterised by HbA_1c_, which was measured in EDTA (edetic acid) whole blood on a calibrated high-performance liquid chromatography system with automated haemolysis before injection. We used mean values from 2002–04 and 2007–09 measurements to represent glycaemic control during follow-up.

We used a comprehensive battery of cognitive tests appropriate for middle-aged individuals.^18^ Short-term verbal memory was tested with a 20-word free-recall test in which participants were presented a list of 20 one-syllable or two-syllable words at intervals of 2 s and were asked to recall in writing as many of the words as possible, in any order, in 2 min. We used the Alice Heim 4-I test^19^ to assess inductive reasoning, measuring the ability of participants to identify patterns and infer principles and rules. This test consists of a series of 65 verbal and mathematical reasoning items of increasing difficulty; participants had 10 min to do the test. Verbal fluency was assessed with tests of phonemic fluency (words beginning with s) and semantic fluency (animal names),^20^ with 1 min allowed for each test. We standardised the results of the four tests to *Z* scores using the mean and SD from the 1997–99 assessments, and averaged them to create a global cognitive score. The global cognitive score was then re-standardised so that the mean was 0 and the SD was 1. Previous research has used global scores constructed in this way to minimise problems caused by measurement error in the individual tests^21^ and to allow comparison of findings across studies when effects are not limited to one cognitive domain. For all cognitive tests, including the global score, we used standardised values in the regression analysis to allow comparison of beta coefficients between results for the different tests.

Covariates included sociodemographic characteristics, health-related behaviours, and chronic diseases. Sociodemographic characteristics were age, sex, marital status (single, divorced, widowed, married, or cohabiting) and education (low [did not complete secondary school], middle [secondary school], and high [university degree or higher]). Health-related behaviours were smoking (current smoker, ex-smoker, or never smoked), alcohol consumption per week (abstainer [zero units], moderate drinker [one to 21 units for men, one to 14 units for women], or heavy drinker [more than 21 units for men, more than 14 units for women]), adequate physical activity (yes or no; as per WHO recommendations,^22^ assessed via a 20-item questionnaire); and frequency of fruit and vegetable consumption (less than once daily, once daily, or more frequently). Chronic disease covariates (based on self-report of doctor diagnosis and corroborated in medical records) were coronary heart disease, stroke, hypertension (defined as blood pressure of 140/90 mm Hg or higher, or use of antihypertensive drugs), respiratory disease, total cholesterol, obesity (BMI of 30 kg/m^2^ or greater), use of antidepressants, and use of lipid-lowering drugs.

### Statistical analysis

We examined associations between baseline characteristics and diabetes status using χ^2^ squared tests (categorical data) and analysis of variance (continuous data). We did cross-sectional analyses for diabetes status and cognitive measures from 1997–99 using linear regression. We used three sets of adjustments with measures from 1997–99: model 1 was adjusted for sociodemographic measures only; model 2 also included health-related behaviours; and model 3 also included chronic diseases.

For longitudinal analyses, we used linear mixed models to examine the association of diabetes status in 1997–99 with cognitive decline over 10 years (1997–99, 2002–04, and 2007–09). In these models, fixed effects were terms for time, main effect terms for diabetes status and all covariates (main effects for covariates allow for adjustment of their cross-sectional effect on cognitive function), and interactions between time and all variables in the model. The interaction between a variable and time represents its effect on cognitive decline, and inclusion of interactions with time for all covariates allows the estimation of diabetes and cognitive decline to be adjusted for the effect of all covariates on cognitive decline. Both the intercept and the slope were fitted as random effects, allowing individuals to have different cognitive scores at baseline and different rates of cognitive decline during follow-up.

Age was centred at the median on the basis of the 1997–99 assessment and used as the timescale in the longitudinal analyses. This term was divided by ten; thus, the coefficient associated with unit change in time represents cognitive decline over 10 years to match the 10 year follow-up. These analyses were also adjusted for age at the start of the cognitive follow-up and included covariates from 1997–99 across the three models, as in the cross-sectional analyses. The normoglycaemia group was used as the reference group to calculate differences in cognitive decline in the prediabetes and diabetes groups.

To allow interpretation of the cross-sectional and longitudinal estimates, we compared them with the effect of age on cognition by dividing the estimate by the effect of a 1 year increase in age on cognition. We calculated the effect of age by regression of the standardised 1997–99 cognitive score on age.

In the final analyses, we examined whether poorer glycaemic control, modelled as a one percentage point increment in HbA_1c_, was associated with cognitive decline in participants with normoglycaemia, prediabetes, newly diagnosed diabetes, or known diabetes. The normal distribution of the HbA_1c_ measure allowed the use of a one percentage point increment as the exposure in these analyses, which were done by use of linear mixed models with an interaction term between diabetes status, HbA_1c_, and time of follow-up to estimate cognitive decline associated with a one percentage point increase in HbA_1c_.

Non-white participants (n=502) were excluded from the main analyses because a test of interaction showed the association of diabetes with cognition to differ in this subgroup (p=0·0013 for the global cognitive score), and small numbers precluded further analyses. However, to allow comparison, we examined cognitive decline as a function of diabetes status (yes or no) in these participants. We also did sensitivity analyses to assess the robustness of our main findings. These were: replacing the hypertension measure in model 3 with systolic and diastolic blood pressure as continuous variables; removing from the analysis all participants who became diabetic after the clinical examination in 1997–99, based on clinical assessments in 2002–04 and 2007–09; using an alternative classification of diabetes status in which newly diagnosed diabetes and known diabetes were replaced with diabetes diagnosis 0–1·5 years ago and diabetes diagnosis more than 1·5 years ago, respectively, on the basis of age at diagnosis of diabetes and age at the 1997–99 clinical assessment; using all covariates as time-dependent variables; and using a multiple imputation, chained-equations method to replace missing data for cognition and covariates during follow-up, using all available data for exposures, outcomes, and covariates in the analysis. All analyses were done in Stata SE version 12 for Windows (StataCorp, 2011). p values were two sided and p<0·05 was regarded as significant.

### Role of the funding source

The sponsors of the study had no role in study design, data collection, data analysis, data interpretation, or writing of the report. The corresponding author had full access to all the data in the study and had final responsibility for the decision to submit for publication.

## Results

Of the 10 308 participants recruited at the beginning of the study in 1985–88, 8637 (84%) attended the diabetes screening in 1991–93, and 7870 (76%) attended in 1997–99 when the first cognitive assessment took place (figure). The median age of participants was 54·4 years (IQR 50·3–60·3) in 1997–99, 59·9 years (55·8–65·7) in 2002–04, and 64·7 years (60·8–70·6) in 2007–09. No differences in sex (interaction p=0·15–0·97) or age (interaction p=0·39–0·77) were noted in associations of diabetes with cognitive decline, leading us to combine men and women and all age groups in the analyses and to adjust the models for age and sex.

**Figure:**
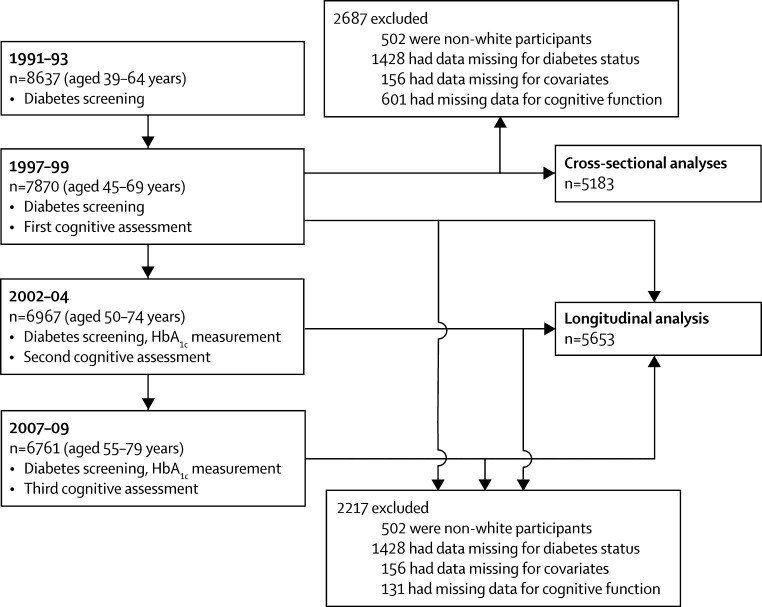
Study profile

Table 1 shows the characteristics of the study population by diabetes status in 1997–99. The mean duration of diabetes in participants with known diabetes was 4·95 (SD 2·21) years. Of the 5653 people included in the longitudinal analyses, 4073 (72%) had cognitive data recorded at all three assessments, 1000 (18%) at two assessments, and 580 (10%) at one assessment. Compared with individuals not included in the longitudinal analyses, the samples consisted of younger participants (mean age 55·5 *vs* 57·1 years, p<0·0001), and contained more men (4113 [73%] of 5653 *vs* 1360 [61%] of 2217, p<0·0001), and more educated individuals (1678 [30%] of 5653 *vs* 542 [24%] of 2217 with a university degree, p<0·0001).

**Table 1:** Characteristics of the study population, by diabetes status in 1997–99

	**Normoglycaemia (n=4703)**	Prediabetes*(n=648)	**Newly diagnosed diabetes (n=115)**	**Known diabetes (n=187)**	**p value**
Age (years)	55·1 (5·9)	57·5 (6·1)	59·0 (6·1)	57·4 (6·3)	<0·0001
Men	3428 (73%)	474 (73%)	81 (70%)	130 (70%)	0·71
Single, divorced, or widowed	1106 (24%)	146 (23%)	32 (28%)	47 (25%)	0·61
Low education	2018 (43%)	292 (45%)	56 (49%)	93 (50%)	0·14
Heavy alcohol consumption†	1270 (27%)	195 (30%)	26 (23%)	52 (28%)	0·26
Current smoker	482 (10%)	48 (7%)	12 (10%)	14 (7%)	0·10
Inadequate physical activity‡	3522 (75%)	477 (74%)	92 (80%)	153 (82%)	0·08
Fruit and vegetable intake less than once daily	1218 (26%)	144 (22%)	27 (23%)	41 (22%)	0·14
Respiratory illness	351 (7%)	43 (7%)	6 (5%)	20 (11%)	0·23
Total cholesterol (mmol/L)	5·91 (1·05)	6·12 (1·06)	6·12 (1·12)	5·92 (1·03)	<0·0001
Obesity (BMI ≥30 kg/m^2^)	561 (12%)	124 (19%)	20 (17%)	43 (23%)	<0·0001
Hypertension	1134 (24%)	257 (40%)	53 (46%)	88 (47%)	<0·0001
Stroke	14 (<1%)	1 (<1%)	1 (1%)	2 (1%)	0·17
Coronary heart disease	240 (5%)	44 (7%)	14 (12%)	21 (11%)	<0·0001
Use of antidepressant drugs	129 (3%)	12 (2%)	5 (4%)	5 (3%)	0·39
Use of lipid-lowering drugs	122 (3%)	22 (3%)	9 (8%)	14 (7%)	<0·0001

Data are n (%) or mean (SD).

* Prediabetes was defined with a 75 g oral glucose tolerance test as one of two states: impaired fasting glucose, defined as fasting plasma glucose between 6·1 mmol/L and less than 7·0 mmol/L, without impaired glucose tolerance; or impaired glucose tolerance, defined as fasting glucose of less than 7·0 mmol/L and a 2 h postload plasma glucose concentration between 7·8 mmol/L and less than 11·1 mmol/L.

† More than 21 units per week for men and more than 14 units per week for women.

‡ Less than WHO recommendations.^22^

Age was inversely associated with cognition; a 1 year increase in age was associated with a −0·039 SD (95% CI −0·043 to −0·035) decrement in memory, a −0·035 SD (−0·039 to −0·030) decrement in reasoning, a −0·036 SD (−0·041 to −0·032) decrement in phonemic fluency, a −0·040 SD (−0·044 to −0·035) decrement in semantic fluency, and a −0·050 SD (−0·054 to −0·046) decrement in global cognitive score (all p<0·0001).

Cross-sectional analyses were based on 5183 people with complete cognitive data in 1997–99; 606 (12%) had prediabetes, 110 (2%) had newly diagnosed diabetes, and 146 (3%) had known diabetes. Compared with normoglycaemic individuals, those with known diabetes had a −0·16 SD (95% CI −0·30 to −0·02) lower score in reasoning in the fully adjusted model (model 3; p=0·023; table 2), although the results for the other cognitive measures (including global cognitive score) were not significant. The coefficient from model 3 in individuals with known diabetes corresponds to an age effect of roughly 4·6 years for reasoning.

**Table 2:** Estimated differences in cognitive function, as a function of diabetes status (cross-sectional analysis at 1997–99 assessment)

	**Model 1**	**Model 2**	**Model 3**
**Memory**			
Normoglycaemia	..	..	..
Prediabetes	−0·05 (−0·13 to 0·03)	−0·05 (−0·13 to 0·03)	−0·04 (−0·13 to 0·04)
Newly diagnosed diabetes	−0·09 (−0·27 to 0·09)	−0·08 (−0·26 to 0·10)	−0·07 (−0·25 to 0·12)
Known diabetes	0·01 (−0·15 to 0·17)	0·02 (−0·14 to 0·17)	0·03 (−0·13 to 0·18)
**Reasoning**			
Normoglycaemia	..	..	..
Prediabetes	0·03 (−0·04 to 0·10)	0·02 (−0·05 to 0·09)	0·03 (−0·04 to 0·10)
Newly diagnosed diabetes	0·05 (−0·12 to 0·21)	0·06 (−0·10 to 0·22)	0·07 (−0·09 to 0·23)
Known diabetes	−0·19 (−0·33 to −0·05)*	−0·18 (−0·32 to −0·04)†	−0·16 (−0·30 to −0·02)‡
**Phonemic fluency**			
Normoglycaemia	..	..	..
Prediabetes	−0·05 (−0·13 to 0·03)	−0·06 (−0·14 to 0·02)	−0·05 (−0·13 to 0·03)
Newly diagnosed diabetes	−0·03 (−0·21 to 0·15)	−0·02 (−0·20 to 0·16)	−0·01 (−0·18 to 0·17)
Known diabetes	−0·07 (−0·23 to 0·08)	−0·07 (−0·23 to 0·08)	−0·05 (−0·21 to 0·10)
**Semantic fluency**			
Normoglycaemia	..	..	..
Prediabetes	−0·03 (−0·11 to 0·05)	−0·03 (−0·11 to 0·05)	−0·03 (−0·11 to 0·05)
Newly diagnosed diabetes	−0·09 (−0·26 to 0·09)	−0·08 (−0·25 to 0·10)	−0·07 (−0·24 to 0·11)
Known diabetes	−0·13 (−0·28 to 0·02)	−0·12 (−0·27 to 0·03)	−0·11 (−0·27 to 0·04)
**Global score**			
Normoglycaemia	..	..	..
Prediabetes	−0·03 (−0·11 to 0·04)	−0·04 (−0·12 to 0·03)	−0·03 (−0·11 to 0·04)
Newly diagnosed diabetes	−0·05 (−0·22 to 0·11)	−0·04 (−0·21 to 0·12)	−0·02 (−0·19 to 0·14)
Known diabetes	−0·13 (−0·28 to 0·01)	−0·12 (−0·26 to 0·02)	−0·10 (−0·25 to 0·04)

Data are beta coefficients (95% CI) based on standardised cognitive scores (mean =0, SD=1). p values for significant results (p<0·05) compared with reference group are indicated by footnotes. Model 1 is adjusted for age, sex, marital status, and education. Model 2 is adjusted for same parameters as model 1 plus health-related behaviours (smoking, alcohol, physical activity, and fruit and vegetable consumption). Model 3 is adjusted for same parameters as model 2 plus coronary heart disease, stroke, hypertension, respiratory disease, total cholesterol, obesity, use of antidepressants, and use of lipid-lowering drugs. n for normoglycaemia is 4321; n for prediabetes is 606; n for newly diagnosed diabetes is 110; and n for known diabetes is 146.

* p=0·008.

† p=0·011.

‡ p=0·023.

We initially did the longitudinal analyses using a simple binary classification of diabetes status in 1997–99: diabetic versus non-diabetic (normoglycaemia and prediabetes). These results (appendix p 1) show faster declines in reasoning, phonemic fluency, and the global cognitive score in participants with diabetes than in those without diabetes. The effect sizes were larger, albeit with wide CIs, in non-white participants (appendix p 2).

Estimates for decline in the normoglycaemic group—used as a reference in the analyses—are listed in the appendix (p 10). Compared with normoglycaemic participants, those with known diabetes had a 45% faster decline in memory, a 29% faster decline in reasoning, and a 24% faster decline in global cognition; compared with the reference values, 10 year differences in decline were −0·13 SD (−0·26 to −0·00) for memory; −0·10 SD (−0·19 to −0·01) for reasoning, and −0·11 SD (−0·21 to −0·02) for the global cognitive score in model 3 (table 3). The significant decline in participants with known diabetes was equivalent to an age effect of 3·3 years for memory, 2·9 years for reasoning, and 2·2 years for the global cognitive score. Participants with prediabetes and newly diagnosed diabetes did not show faster cognitive decline than those with normoglycaemia (table 3).

**Table 3:** Estimated differences in cognitive decline over 10 years, as a function of diabetes status in 1997–99

	**Model 1**	**Model 2**	**Model 3**
**Memory**			
Normoglycaemia	..	..	..
Prediabetes	0·02 (−0·05 to 0·09)	0·02 (−0·05 to 0·09)	0·02 (−0·05 to 0·09)
Newly diagnosed diabetes	0·07 (−0·09 to 0·22)	0·06 (−0·10 to 0·21)	0·06 (−0·10 to 0·21)
Known diabetes	−0·13 (−0·26 to −0·01)*	−0·13 (−0·26 to −0·00)†	−0·13 (−0·26 to −0·00)‡
**Reasoning**			
Normoglycaemia	..	..	..
Prediabetes	−0·02 (−0·07 to 0·02)	−0·03 (−0·07 to 0·02)	−0·02 (−0·07 to 0·02)
Newly diagnosed diabetes	−0·04 (−0·14 to 0·06)	−0·04 (−0·15 to 0·06)	−0·05 (−0·15 to 0·06)
Known diabetes	−0·10 (−0·19 to −0·01)§	−0·10 (−0·18 to −0·01)¶	−0·10 (−0·19 to −0·01)‖
**Phonemic fluency**			
Normoglycaemia	..	..	..
Prediabetes	0·02 (−0·04 to 0·08)	0·02 (−0·04 to 0·07)	0·02 (−0·04 to 0·08)
Newly diagnosed diabetes	−0·10 (−0·23 to 0·04)	−0·10 (−0·24 to 0·03)	−0·10 (−0·23 to 0·04)
Known diabetes	−0·09 (−0·20 to 0·02)	−0·08 (−0·20 to 0·03)	−0·08 (−0·19 to 0·03)
**Semantic fluency**			
Normoglycaemia	..	..	..
Prediabetes	0·00 (−0·05 to 0·06)	0·00 (−0·06 to 0·06)	0·00 (−0·05 to 0·06)
Newly diagnosed diabetes	0·03 (−0·10 to 0·16)	0·03 (−0·11 to 0·16)	0·02 (−0·11 to 0·16)
Known diabetes	−0·06 (−0·17 to 0·05)	−0·05 (−0·16 to 0·06)	−0·05 (−0·16 to 0·06)
**Global score**			
Normoglycaemia	..	..	..
Prediabetes	−0·00 (−0·05 to 0·05)	−0·00 (−0·05 to 0·05)	−0·00 (−0·05 to 0·05)
Newly diagnosed diabetes	−0·05 (−0·16 to 0·06)	−0·05 (−0·16 to 0·06)	−0·05 (−0·16 to 0·06)
Known diabetes	−0·12 (−0·21 to −0·02)**	−0·11 (−0·21 to − 0·02)††	−0·11 (−0·21 to −0·02)‡‡

Data are beta coefficients (95% CI) based on standardised cognitive scores (mean=0, SD=1); −0·00 occurs because of rounding. Longitudinal analyses are based on data for cognitive function from 1997–99, 2002–04, 2007–09. p values for significant results (p<0·05) compared with reference group are indicated by footnotes. Model 1 is adjusted for age, sex, marital status, and education. Model 2 is adjusted for same parameters as model 1 plus health-related behaviours (smoking, alcohol, physical activity, and fruit and vegetable consumption). Model 3 is adjusted for same parameters as model 2 plus coronary heart disease, stroke, hypertension, respiratory disease, total cholesterol, obesity, use of antidepressants, and use of lipid-lowering drugs. n for normoglycaemia is 4703; n for prediabetes is 648; n for newly diagnosed diabetes is 115; and n for known diabetes is 187.

* p=0·039.

† p=0·042.

‡ p=0·046.

§ p=0.028.

¶ p=0·028.

‖ p=0·026.

** p=0·014.

†† p=0·015.

‡‡ p=0·014.

Sensitivity analyses showed that replacing hypertension with systolic and diastolic blood pressure as continuous variables in model 3 had little effect on the estimates (appendix p 3); the results were much the same when participants who became diabetic during the period of cognitive testing were removed from the analysis (appendix p 4). Alternative classification of diabetes status (diagnosed 0–1·5 years ago *vs* more than 1·5 years ago) showed a faster cognitive decline in participants who had been diagnosed with diabetes more than 1·5 years ago than in those diagnosed more recently (appendix p 5). Use of time-dependent covariates in the longitudinal models showed significantly faster decline in reasoning and the global cognitive score in those with known diabetes (appendix p 6). Associations using imputed data showed stronger cross-sectional effects (appendix p 7), but longitudinal results were similar to those from the main analysis (appendix p 8).

Mean HbA_1c_ values were highest in participants with known diabetes (6·84%, SD 1·25) and lowest in those with normoglycaemia (5·40%, 0·40); newly diagnosed individuals (6·27%, 1·10) and participants with prediabetes (5·71%, 0·65) had intermediate values. In the fully adjusted analyses (model 3), a one percentage point increment in HbA_1c_ was associated with a significantly faster decline in memory in participants with known diabetes, and a faster decline in reasoning in those with newly diagnosed (significant [p=0·028]) and known diabetes (approaching significance [p=0·052]; table 4). Using time-dependent covariates rather than those drawn from the 1997–99 assessment showed similar associations (appendix p 9).

**Table 4:** Association of glycaemic control (one percentage point increment in HbA_1c_) with estimated differences in cognitive decline, by diabetes status in 1997–99

	**Model 1**	**Model 2**	**Model 3**
**Memory**			
Normoglycaemia	−0·02 (−0·09 to 0·04)	−0·03 (−0·09 to 0·03)	−0·02 (−0·08 to 0·04)
Prediabetes	−0·06 (−0·15 to 0·04)	−0·05 (−0·15 to 0·04)	−0·05 (−0·15 to 0·04)
Newly diagnosed diabetes	−0·09 (−0·24 to 0·06)	−0·09 (−0·23 to 0·06)	−0·09 (−0·23 to 0·06)
Known diabetes	−0·13 (−0·23 to −0·02)*	−0·12 (−0·23 to −0·02)†	−0·12 (−0·22 to −0·01)‡
**Reasoning**			
Normoglycaemia	−0·02 (−0·06 to 0·02)	−0·01 (−0·05 to 0·03)	−0·02 (−0·06 to 0·02)
Prediabetes	−0·01 (−0·07 to 0·06)	−0·00 (−0·07 to 0·06)	0·00 (−0·07 to 0·06)
Newly diagnosed diabetes	−0·10 (−0·20 to −0·01)§	−0·11 (−0·20 to −0·01)¶	−0·11 (−0·20 to −0·01)‖
Known diabetes	−0·07 (−0·15 to −0·00)**	−0·08 (−0·15 to −0·00)††	−0·07 (−0·15 to 0·00)
**Phonemic fluency**			
Normoglycaemia	−0·03 (−0·08 to 0·03)	−0·02 (−0·07 to 0·03)	−0·01 (−0·07 to 0·04)
Prediabetes	−0·07 (−0·15 to 0·01)	−0·07 (−0·15 to 0·02)	−0·06 (−0·14 to 0·02)
Newly diagnosed diabetes	−0·06 (−0·19 to 0·06)	−0·07 (−0·19 to 0·06)	−0·07 (−0·19 to 0·06)
Known diabetes	0·01 (−0·09 to 0·11)	0·00 (−0·09 to 0·10)	0·01 (−0·09 to 0·10)
**Semantic fluency**			
Normoglycaemia	0·01 (−0·04 to 0·06)	0·01 (−0·04 to 0·07)	0·01 (−0·04 to 0·06)
Prediabetes	0·01 (−0·07 to 0·09)	0·01 (−0·07 to 0·09)	0·01 (−0·07 to 0·09)
Newly diagnosed diabetes	0·02 (−0·10 to 0·15)	0·02 (−0·11 to 0·14)	0·02 (−0·11 to 0·14)
Known diabetes	−0·02 (−0·11 to 0·08)	−0·02 (−0·11 to 0·07)	−0·02 (−0·12 to 0·07)
**Global score**			
Normoglycaemia	−0·01 (−0·06 to 0·03)	−0·01 (−0·05 to 0·03)	−0·01 (−0·05 to 0·03)
Prediabetes	−0·05 (−0·11 to 0·02)	−0·04 (−0·11 to 0·02)	−0·04 (−0·11 to 0·03)
Newly diagnosed diabetes	−0·08 (−0·18 to 0·02)	−0·08 (−0·18 to 0·02)	−0·08 (−0·18 to 0·02)
Known diabetes	−0·06 (−0·14 to 0·02)	−0·07 (−0·15 to 0·01)	−0·06 (−0·14 to 0·01)

Data are beta coefficients (95% CI) based on standardised cognitive scores (mean=0, SD=1); −0·00 occurs because of rounding. Data for cognitive function are from 1997–99, 2002–04, and 2007–09. p values for significant results (p<0·05) compared with reference group are indicated by footnotes. Model 1 is adjusted for age, sex, marital status, and education. Model 2 is adjusted for same parameters as model 1 and health-related behaviours (smoking, alcohol, physical activity, and fruit and vegetable consumption). Model 3 is adjusted for same parameters as model 2 and coronary heart disease, stroke, hypertension, respiratory disease, total cholesterol, obesity, use of antidepressants, and use of lipid-lowering drugs. n for normoglycaemia is 4336; n for prediabetes is 572; n for newly diagnosed diabetes is 100; and n for known diabetes is 152.

* p=0·020.

† p=0·022.

‡ =0·034.

§ p=0·034.

¶ p=0·027.

‖ p=0·028.

** p=0·049.

†† p=0·040.

## Discussion

Our results from a large cohort of middle-aged adults show that participants with known diabetes—ie, those who had diabetes at the start of the study—had an increased rate of cognitive decline during the subsequent 10 year period. The effect of diabetes duration cannot be examined when all diabetes cases are analysed together, showing the pertinence of our research design. Faster cognitive decline in people with long-term diabetes, taking into account both cross-sectional and longitudinal analyses, corresponded to an age effect of 7·5 years for reasoning and roughly 4·4 years for the global cognitive score. Cognitive decline in those with newly diagnosed diabetes and prediabetes was not different from that which occurred in normoglycaemic participants. We noted little attenuation of associations after taking into account potential confounding factors. Our results also show that in people with diabetes, those with poorer glycaemic control had faster cognitive decline. These findings suggest that duration of diabetes contributes to faster cognitive decline and that good glycaemic control could decrease this risk (panel).

Panel **Research in context**

**Systematic review**

We searched PubMed for research articles and reviews in English published up to Sept 30, 2013. We used the search terms “type 2 diabetes” and “cognitive decline” in the title or abstract. We also searched the reference lists of retrieved articles and identified additional relevant publications on the link between diabetes, cognitive deficits, and dementia through manual search. We found consistent evidence that the risk of dementia is increased in people with type 2 diabetes.^2^, ^3^, ^4^ However, previous studies did not closely examine the relation between diabetes duration and cognitive decline, or the effect of glycaemic control.

**Interpretation**

Our results show that a longer diabetes duration is associated with faster cognitive decline. Additionally, for people with diabetes, poor glycaemic control was associated with faster cognitive decline. Thus, interventions that delay diabetes onset, as well as tight glycaemic control in those with established disease, might help to prevent some of the deleterious effects of type 2 diabetes on cognitive ageing.

Substantial evidence suggests that diabetes is a risk factor for cognitive decline and dementia.^2^, ^3^ Some previous studies have suggested that the faster rate of cognitive decline affects only elderly people with type 2 diabetes,^23^ with the hypothesis being that type 2 diabetes does not affect cognition before old age.^10^, ^11^ However, our results, from participants with median ages of 54, 60, and 65 years at the time of cognitive assessments, show that longer duration of diabetes is associated with more rapid cognitive decline. These findings are in line with previous results suggesting that midlife rather than late-life diabetes is a risk factor for dementia.^24^ These, along with our results, can be interpreted as showing that longer exposure to diabetes is harmful for cognition. A previous case-control study^25^ in elderly people showed duration and severity of diabetes to be associated with mild cognitive impairment. Another study^9^ in adults aged 40–83 years, who were followed up for 12 years, showed that the extent of cognitive decline in individuals who developed diabetes during follow-up was between that of individuals without diabetes and those who had diabetes at baseline, albeit not significantly different from either group. Although our finding that cognitive decline was not worse in those with prediabetes is in agreement with that of a previous study^26^ in elderly women, further research is needed since high glucose concentrations have been associated with an increased risk of dementia in people with glucose concentrations below the clinical threshold of manifest diabetes.^27^

All cognitive tests used in our analyses have been shown previously to be sensitive to age-related changes in cognition in midlife.^18^ In our study, known diabetes was associated with faster decline in memory, reasoning, and the global cognitive score. Although no significant cross-sectional effects were evident for memory, the memory decline over 10 years was 45% faster in participants with known diabetes. Additionally, poor glycaemic control in diabetes was associated with a faster decline in memory and possibly in reasoning. Thus, various components of cognition seem to be affected by type 2 diabetes, as evident in the robust effects on the global cognitive score.

The precise mechanisms that underlie the association of diabetes with cognitive decline and dementia remain unclear. Vascular pathways are considered to be important.^3^ Diabetes is often associated with common cardiovascular risk factors such as dyslipidaemia, hypertension, and obesity.^28^ Complications related to microangiopathy have also been implicated.^29^ Heterogeneous cerebral lesions, which cause cognitive dysfunction, are associated with longer diabetes duration; these lesions include ischaemic stroke, intracerebral haemorrhage, lacunar infarcts, white matter lesions, and cerebral atrophy.^30^

Our results emphasise the importance of duration of diabetes for cognitive ageing, and suggest that interventions that aim to prevent or delay diabetes onset might have implications for cognitive health. Lifestyle interventions have been shown to reduce the risk of progression from prediabetes to diabetes. A randomised trial^31^ of adults at high risk of type 2 diabetes showed that an intensive lifestyle modification programme reduced the risk of progression to diabetes by more than use of the antidiabetic drug metformin (58% [95 % CI 48–66] *vs* 31% [17–43]). Less clear is the effect of tight glycaemic control on those with established disease. In the ACCORD MIND study,^32^ intervention to reduce HbA_1c_ to less than 6% in people with type 2 diabetes was associated with reduced brain atrophy, although no effect on cognitive decline was evident. By contrast, in the IDEATel trial,^33^ an HbA_1c_ of 7% or less was associated with slowed cognitive decline. Prevention of microvascular complications is highly dependent on glycaemic control in adults with type 2 diabetes, but no effect on macrovascular disease or mortality was seen in elderly people with longstanding type 2 diabetes.^34^ Cumulative glycaemic exposure (ie, severity and duration of hyperglycaemia) is important for microvascular complications,^35^ and increases the risk of more rapid cognitive decline.

The strengths of this analysis from the Whitehall II study are the prospective cohort and the fairly young population—75% of participants were younger than 71 years at the last cognitive assessment. The repeated standardised screening for diabetes before the start of cognitive follow-up allowed us to minimise reverse causation biases. Alternative classification of duration of diabetes gave similar results. We also took into account a range of potential confounders of the association between diabetes and cognition. The major limitation of this study is the issue of generalisability, since the data are for an occupational cohort and the participants are likely to be healthier than the general population. Finally, because of the small numbers of non-white participants, we could not examine the duration-of-diabetes hypothesis in this group, so the extent to which the results apply to non-white populations is unclear.

Our results support the hypothesis that the risk of accelerated cognitive decline in people with diabetes depends on how long an individual has had the disease and on the extent to which they can achieve normal carbohydrate metabolism. Further research is needed to determine whether improving management of type 2 diabetes also reduces the risk of dementia.
