# Supplementary material for: Midlife type 2 diabetes and poor glycaemic control as risk factors for cognitive decline in early old age: a post-hoc analysis of the Whitehall II cohort study
Source: Lancet Diabetes Endocrinol. 2014 Mar 6;2(3):228–35. doi: 10.1016/S2213-8587(13)70192-X (PMC4274502; doi:10.1016/S2213-8587(13)70192-X)
Supplement: Supplementary appendix [file mmc1.pdf]

THE LANCET Diabetes & Endocrinology

Supplementary appendix

This appendix formed part of the original submission and has been peer reviewed. We post it as supplied by the authors.

Supplement to: Tuligenga RH, Dugravot A, Tabák AG, et al. Midlife type 2 diabetes and poor glycaemic control as risk factors for cognitive decline in early old age: a post-hoc analysis of the Whitehall II cohort study. *Lancet Diabetes Endocrinol* 2013; published online Dec 19. http://dx.doi.org/10.1016/S2213-8587(13)70192-X.

***Table e1:* Estimated difference in cognitive decline over 10 years in white participants, as a function of type 2 diabetes in 1997–99**

	**Model 1**	**Model 2**	**Model 3**
**Memory**			
No diabetes	..	..	..
Type 2 diabetes	-0·06 (-0·15 to 0·04)	-0·06 (-0·15 to 0·04)	-0·05 (-0·15 to 0·04)
**Reasoning**			
No diabetes	..	..	..
Type 2 diabetes	-0·07 (-0·14 to -0·00)*	-0·07 (-0·14 to -0·00)†	-0·07 (-0·14 to -0·00)‡
**Phonemic fluency**			
No diabetes	..	..	..
Type 2 diabetes	-0·10 (-0·18 to -0·01)§	-0·09 (-0·18 to -0·01)¶	-0·09 (-0·17 to 0·00)‖
**Semantic fluency**			
No diabetes	..	..	..
Type 2 diabetes	-0·02 (-0·11 to 0·07)	-0·02 (-0·10 to 0·07)	-0·02 (-0·11 to 0·07)
**Global score**			
No diabetes	..	..	..
Type 2 diabetes	-0·08 (-0·16 to -0·01)**	-0·09 (-0·16 to -0·01)††	-0·09 (-0·16 to -0·02)‡‡

Data are beta coefficients (95% CI) based on standardised cognitive scores (mean=0, SD=1); –0·00 occurs because of rounding. Data for cognitive function are from 1997–99, 2002–04, and 2007–09. p values for significant results (p<0·05) compared with reference group are indicated by footnotes. Model 1 is adjusted for age, sex, marital status, and education. Model 2 is adjusted for same parameters as model 1 and health-related behaviours (smoking, alcohol, physical activity, and fruit and vegetable consumption). Model 3 is adjusted for same parameters as model 2 and coronary heart disease, stroke, hypertension, respiratory disease, total cholesterol, obesity, use of antidepressants, and use of lipid-lowering drugs. n for no diabetes is 5351; n for type 2 diabetes is 302. *p=0·048. †p=0·045. ‡p=0·038. §p=0·031. ¶p=0·037. ‖p=0·049. **p=0·020. ††p=0·019. ‡‡p=0·017.

Table e2: **Estimated difference in cognitive decline over 10 years in non-white participants, as a function of type 2 diabetes in 1997–99**

	**Model 1**	**Model 2**	**Model 3**
**Memory**			
No diabetes	··	··	··
Type 2 diabetes	-0·12 (-0·33 to 0·08)	-0·15 (-0·36 to 0·05)	-0·15 (-0·36 to 0·07)
**Reasoning**			
No diabetes	··	··	··
Type 2 diabetes	0·04 (-0·10 to 0·17)	0·04 (-0·09 to 0·18)	0·04 (-0·10 to 0·18)
**Phonemic fluency**			
No diabetes	··	··	··
Type 2 diabetes	-0·15 (-0·33 to 0·04)	-0·16 (-0·35 to 0·02)	-0·19 (-0·38 to 0·00)
**Semantic fluency**			
No diabetes	··	··	··
Type 2 diabetes	-0·02 (-0·20 to 0·16)	-0·01 (-0·18 to 0·17)	0·01 (-0·18 to 0·20)
**Global score**			
No diabetes	··	··	··
Type 2 diabetes	-0·03 (-0·26 to 0·20)	-0·04 (-0·28 to 0·19)	-0·05 (-0·29 to 0·19)

Data are beta coefficients (95% CI) based on standardised cognitive scores (mean=0, SD=1). Data for cognitive function are from 1997–99, 2002–04, and 2007–09. Model 1 is adjusted for age, sex, marital status, and education. Model 2 is adjusted for same parameters as model 1 and health-related behaviours (smoking, alcohol, physical activity, and fruit and vegetable consumption). Model 3 is adjusted for same parameters as model 2 and coronary heart disease, stroke, hypertension, respiratory disease, total cholesterol, obesity, use of antidepressants, and use of lipid-lowering drugs. n for no diabetes is 409; n for type 2 diabetes is 93.

Table e3: **Estimated difference in cognitive decline over 10 years, as a function of diabetes status in 1997–99 (with systolic and diastolic blood pressure used as continuous variables in model 3)**

	**Model 1**	**Model 2**	**Model 3**
**Memory**			
Normoglycaemia	··	··	··
Prediabetes	0·02 (-0·05 to 0·09)	0·02 (-0·05 to 0·09)	0·02 (-0·05 to 0·09)
Newly diagnosed diabetes	0·07 (-0·09 to 0·22)	0·06 (-0·10 to 0·21)	0·05 (-0·11 to 0·21)
Known diabetes	-0·13 (-0·26 to -0·01)*	-0·13 (-0·26 to -0·00)†	-0·13 (-0·25 to -0·00)‡
**Reasoning**			
Normoglycaemia	··	··	··
Prediabetes	-0·02 (-0·07 to 0·02)	-0·03 (-0·07 to 0·02)	-0·03 (-0·07 to 0·02)
Newly diagnosed diabetes	-0·04 (-0·14 to 0·06)	-0·04 (-0·15 to 0·06)	-0·04 (-0·15 to 0·06)
Known diabetes	-0·10 (-0·19 to -0·01)§	-0·10 (-0·18 to -0·01)¶	-0·10 (-0·19 to -0·01)‖
**Phonemic fluency**			
Normoglycaemia	··	··	··
Prediabetes	0·02 (-0·04 to 0·08)	0·02 (-0·04 to 0·07)	0·02 (-0·04 to 0·08)
Newly diagnosed diabetes	-0·10 (-0·23 to 0·04)	-0·10 (-0·24 to 0·03)	-0·10 (-0·23 to 0·04)
Known diabetes	-0·09 (-0·20 to 0·02)	-0·08 (-0·20, 0·03)	-0·08 (-0·19 to 0·03)
**Semantic fluency**			
Normoglycaemia	··	··	··
Prediabetes	0·00 (-0·05 to 0·06)	0·00 (-0·05 to 0·06)	0·00 (-0·06 to 0·06)
Newly diagnosed diabetes	0·03 (-0·10 to 0·16)	0·03 (-0·11 to 0·16)	0·02 (-0·12 to 0·15)
Known diabetes	-0·06 (-0·17 to 0·05)	-0·05 (-0·16 to 0·06)	-0·06 (-0·17 to 0·05)
**Global score**			
Normoglycaemia	··	··	··
Prediabetes	0·00 (-0·05 to 0·05)	-0·00 (-0·05 to 0·05)	-0·00 (-0·05 to 0·05)
Newly diagnosed diabetes	-0·05 (-0·16 to 0·06)	-0·05 (-0·16 to 0·06)	-0·06 (-0·17 to 0·02)
Known diabetes	-0·12 (-0·21 to -0·02)**	-0·11 (-0·21 to -0·02)††	-0·12 (-0·21 to -0·02)‡‡

Data are beta coefficients (95% CI) based on standardised cognitive scores (mean=0, SD=1); –0·00 occurs because of rounding. Data for cognitive function are from 1997–99, 2002–04, and 2007–09. p values for significant results (p<0·05) compared with reference group are indicated by footnotes. Model 1 is adjusted for age, sex, marital status, and education. Model 2 is adjusted for same parameters as model 1 and health-related behaviours (smoking, alcohol, physical activity, and fruit and vegetable consumption). Model 3 is adjusted for same parameters as model 2 and coronary heart disease, stroke, hypertension, respiratory disease, total cholesterol, obesity, use of antidepressants, and use of lipid-lowering drugs. n for normoglycaemia is 4703; n for prediabetes is 648; n for newly diagnosed diabetes is 115; and n for known diabetes is 187. *p=0·039. †p=0·042. ‡p=0·046. §p=0·028. ¶p=0·028. ‖p=0·027. **p=0·014. ††p=0·015. ‡‡p=0·014.

***Table e4:* Estimated difference in cognitive decline over 10 years, as a function of diabetes status in 1997–99 (with incident cases of type 2 diabetes after 1997–99 removed from the analyses)**

	Model 1	Model 2	Model 3
**Memory**			
Normoglycaemia	··	··	··
Prediabetes	0·02 (-0·06 to 0·09)	0·01 (-0·06 to 0·09)	0·01 (-0·06 to 0·09)
Newly diagnosed diabetes	0·07 (-0·09 to 0·22)	0·06 (-0·10 to 0·21)	0·06 (-0·10 to 0·21)
Known diabetes	-0·13 (-0·26 to -0·01)*	-0·13 (-0·26 to -0·00)†	-0·12 (-0·25 to -0·00)
**Reasoning**			
Normoglycaemia	··	··	··
Prediabetes	-0·01 (-0·06 to 0·04)	-0·01 (-0·06 to 0·04)	-0·01 (-0·06 to 0·04)
Newly diagnosed diabetes	-0·04 (-0·14 to 0·07)	-0·04 (-0·14 to 0·06)	-0·05 (-0·15 to 0·06)
Known diabetes	-0·10 (-0·18 to -0·01)‡	-0·10 (-0·18 to -0·01)§	-0·10 (-0·18 to -0·01)¶
**Phonemic fluency**			
Normoglycaemia	··	··	··
Prediabetes	0·03 (-0·04 to 0·09)	0·03 (-0·04 to 0·09)	0·03 (-0·03 to 0·10)
Newly diagnosed diabetes	-0·10 (-0·23 to 0·04)	-0·10 (-0·24 to 0·03)	0·10 (-0·23 to 0·04)
Known diabetes	-0·09 (-0·20 to 0·02)	-0·08 (-0·19 to 0·03)	-0·07 (-0·18 to 0·04)
**Semantic fluency**			
Normoglycaemia	··	··	··
Prediabetes	0·00 (-0·06 to 0·06)	0·00 (-0·07 to 0·06)	0·00 (-0·06 to 0·06)
Newly diagnosed diabetes	0·03 (-0·10 to 0·17)	0·04 (-0·10 to 0·17)	0·03 (-0·11 to 0·16)
Known diabetes	-0·05 (-0·16 to 0·06)	-0·05 (-0·16 to 0·06)	-0·05 (-0·16 to 0·06)
**Global score**			
Normoglycaemia	··	··	··
Prediabetes	-0·00 (-0·05 to 0·06)	-0·00 (-0·05 to 0·04)	-0·00 (-0·05 to 0·06)
Newly diagnosed diabetes	-0·04 (-0·15 to 0·07)	-0·05 (-0·16 to 0·06)	-0·05 (-0·16 to 0·06)
Known diabetes	-0·11 (-0·20 to -0·02)‖	-0·11 (-0·20 to -0·02)**	-0·11 (-0·20 to -0·02)††

Data are beta coefficients (95% CI) based on standardised cognitive scores (mean=0, SD=1); –0·00 occurs because of rounding. Model 1 is adjusted for age, sex, marital status, and education. Model 2 is adjusted for same parameters as model 1 and health-related behaviours (smoking, alcohol, physical activity, and fruit and vegetable consumption). Model 3 is adjusted for same parameters as model 2 and coronary heart disease, stroke, hypertension, respiratory disease, total cholesterol, obesity, use of antidepressants, and use of lipid-lowering drugs. n for normoglycaemia is 4622; n for prediabetes is 544; n for newly diagnosed diabetes is 115; and n for known diabetes is 187. *p=0·042. †p=0·043. ‡p=0·030. §p=0·031. ¶p=0·032. ‖p=0·016. **p=0·017. ††p=0·021.

Table e5: **Alternative classification of diabetes status: estimated difference in cognitive decline over 10 years, as a function of diabetes status in 1997–99**

	Model 1	Model 2	Model 3
**Memory**			
Normoglycaemia	··	··	··
Prediabetes	0·02 (-0·05 to 0·09)	0·02 (-0·05 to 0·09)	0·02 (-0·05 to 0·09)
Diabetes for 0-1·5 years	0·05 (-0·09 to 0·20)	0·04 (-0·10 to 0·19)	0·04 (-0·10 to 0·19)
Diabetes for >1·5 years	-0·14 (-0·27 to -0·00)*	-0·13 (-0·26 to -0·00)†	-0·13 (-0·26 to -0·00)
**Reasoning**			
Normoglycaemia	··	··	··
Prediabetes	-0·02 (-0·07 to 0·02)	-0·03 (-0·07 to 0·02)	-0·02 (-0·07 to 0·02)
Diabetes for 0-1·5 years	-0·04 (-0·14 to 0·06)	-0·05 (-0·14 to 0·05)	-0·05 (-0·15 to 0·05)
Diabetes for >1·5 years	-0·10 (-0·19 to -0·01)‡	-0·10 (-0·19 to -0·01)§	-0·10 (-0·19 to -0·01)¶
**Phonemic fluency**			
Normoglycaemia	··	··	··
Prediabetes	0·02 (-0·04 to 0·08)	0·02 (-0·04 to 0·07)	0·02 (-0·04 to 0·08)
Diabetes for 0-1·5 years	-0·14 (-0·26 to -0·01)||	-0·14 (-0·27 to -0·02)**	-0·14 (-0·26 to -0·01)††
Diabetes for >1·5 years	-0·06 (-0·17 to 0·06)	-0·05 (-0·16 to 0·07)	-0·04 (-0·16 to 0·08)
**Semantic fluency**			
Normoglycaemia	··	··	··
Prediabetes	0·00 (-0·05 to 0·06)	0·00 (-0·06 to 0·06)	0·00 (-0·05 to 0·06)
Diabetes for 0-1·5 years	0·04 (-0·08 to 0·17)	0·04 (-0·09 to 0·16)	0·04 (-0·09 to 0·16)
Diabetes for >1·5 years	-0·07 (-0·19 to 0·04)	-0·07 (-0·18 to 0·05)	-0·07 (-0·18 to 0·05)
**Global score**			
Normoglycaemia	··	··	··
Prediabetes	-0·00 (-0·05 to 0·05)	-0·00 (-0·05 to 0·05)	0·00 (-0·05 to 0·05)
Diabetes for 0-1·5 years	-0·06 (-0·16 to 0·05)	-0·06 (-0·16 to 0·04)	-0·06 (-0·17 to 0·04)
Diabetes for >1·5 years	-0·11 (-0·21 to -0·01)‡‡	-0·11 (-0·20 to -0·01)§§	-0·11 (-0·20 to -0·01)¶¶

Data are beta coefficients (95% CI) based on standardised cognitive scores (mean=0, SD=1); –0·00 occurs because of rounding. Model 1 is adjusted for age, sex, marital status, and education. Model 2 is adjusted for same parameters as model 1 and health-related behaviours (smoking, alcohol, physical activity, and fruit and vegetable consumption). Model 3 is adjusted for same parameters as model 2 and coronary heart disease, stroke, hypertension, respiratory disease, total cholesterol, obesity, use of antidepressants, and use of lipid-lowering drugs. n for normoglycaemia is 4703; n for prediabetes is 648; n for diabetes duration ≤1·5 years is 134; and n for diabetes duration >1·5 years is 168. *p=0·042. †p=0·048. ‡p=0·036. §p=0·038. ¶p=0·034. ||p=0·035. **p=0·027. ††p=0·036. ‡‡p=0·026. §§p=0·029. ¶¶p=0·028.

***Table e6:* Estimated difference in cognitive decline over 10 years, as a function of diabetes status in 1997–99 (with all covariates entered as time-dependent variables)**

	Model 1	Model 2	Model 3
**Memory**			
Normoglycaemia	··	··	··
Prediabetes	0·02 (-0·05 to 0·09)	0·02 (-0·05 to 0·09)	0·03 (-0·04 to 0·10)
Newly diagnosed diabetes	0·07 (-0·09 to 0·22)	0·06 (-0·10 to 0·21)	0·08 (-0·08 to 0·23)
Known diabetes	-0·13 (-0·26 to -0·01)*	-0·12 (-0·25 to 0·00)	-0·10 (-0·23 to 0·03)
**Reasoning**			
Normoglycaemia	··	··	··
Prediabetes	-0·02 (-0·07 to 0·02)	-0·02 (-0·07 to 0·02)	-0·03 (-0·07 to 0·02)
Newly diagnosed diabetes	-0·04 (-0·14 to 0·06)	-0·04 (-0·15 to 0·06)	-0·05 (-0·15 to 0·05)
Known diabetes	-0·10 (-0·18 to -0·01)†	-0·10 (-0·18 to -0·01)‡	-0·12 (-0·20 to -0·03)§
**Phonemic fluency**			
Normoglycaemia	··	··	··
Prediabetes	0·02 (-0·04 to 0·08)	0·02 (-0·04 to 0·08)	0·02 (-0·04 to 0·08)
Newly diagnosed diabetes	-0·10 (-0·23 to 0·04)	-0·11 (-0·24 to 0·03)	-0·09 (-0·22 to 0·05)
Known diabetes	-0·09 (-0·20 to 0·02)	-0·09 (-0·20 to 0·03)	-0·08 (-0·19 to 0·03)
**Semantic fluency**			
Normoglycaemia	··	··	··
Prediabetes	0·00 (-0·05 to 0·06)	0·00 (-0·05 to 0·06)	0·00 (-0·06 to 0·06)
Newly diagnosed diabetes	0·03 (-0·10 to 0·16)	0·02 (-0·11 to 0·15)	0·03 (-0·10 to 0·16)
Known diabetes	-0·06 (-0·17 to 0·05)	-0·05 (-0·16 to 0·06)	-0·06 (-0·17 to 0·05)
**Global score**			
Normoglycaemia	··	··	··
Prediabetes	0·00 (-0·05 to 0·05)	-0·00 (-0·05 to 0·05)	0·00 (-0·04 to 0·05)
Newly diagnosed diabetes	-0·05 (-0·16 to 0·06)	-0·06 (-0·17 to 0·05)	-0·04 (-0·15 to 0·07)
Known diabetes	-0·11 (-0·21 to -0·02)¶	-0·11 (-0·20 to -0·02)‖	-0·11 (-0·20 to -0·02)**

Data are beta coefficients (95% CI) based on standardised cognitive scores (mean=0, SD=1); –0·00 occurs because of rounding. p values for significant results (p<0·05) compared with reference group are indicated by footnotes. Model 1 is adjusted for age, sex, marital status, and education. Model 2 is adjusted for same parameters as model 1 and health-related behaviours (smoking, alcohol, physical activity, and fruit and vegetable consumption). Model 3 is adjusted for same parameters as model 2 and coronary heart disease, stroke, hypertension, respiratory disease, total cholesterol, obesity, use of antidepressants, and use of lipid-lowering drugs. n for normoglycaemia is 4703; n for prediabetes is 648; n for newly diagnosed diabetes is 115; and n for known diabetes is 187. *p=0·039. †p=0·031. ‡p=0·030. §p=0·009. ¶p=0·015. ‖p=0·016. **p=0·020.

Table e7: **Estimated difference in cognitive function, as a function of diabetes status (cross-sectional analysis; with multiple imputation for missing data for cognitive tests and covariates)**

	**Model 1**	**Model 2**	**Model 3**
**Memory**			
Normoglycaemia	··	··	··
Prediabetes	-0·06 (-0·14 to 0·02)	-0·06 (-0·14 to 0·02)	-0·05 (-0·13 to 0·03)
Newly diagnosed diabetes	-0·08 (-0·26 to 0·10)	-0·07 (-0·25 to 0·11)	-0·06 (-0·24 to 0·12)
Known diabetes	-0·05 (-0·20 to 0·10)	-0·04 (-0·19 to 0·11)	-0·03 (-0·18 to 0·12)
**Reasoning**			
Normoglycaemia	··	··	··
Prediabetes	0·02 (-0·06 to 0·09)	0·01 (-0·06 to 0·08)	0·02 (-0·05 to 0·09)
Newly diagnosed diabetes	0·01 (-0·15 to 0·17)	0·02 (-0·13 to 0·18)	0·04 (-0·12 to 0·20)
Known diabetes	-0·23 (-0·36 to -0·10)*	-0·22 (-0·35 to -0·09)†	-0·20 (-0·32 to -0·07)‡
**Phonemic fluency**			
Normoglycaemia	··	··	··
Prediabetes	-0·06 (-0·14 to 0·02)	-0·06 (-0·15 to 0·01)	-0·06 (-0·14 to 0·02)
Newly diagnosed diabetes	-0·05 (-0·22 to 0·13)	-0·04 (-0·21 to 0·14)	-0·02 (-0·19 to 0·16)
Known diabetes	-0·11 (-0·25 to 0·04)	-0·10 (-0·25 to 0·04)	-0·08 (-0·22 to 0·06)
**Semantic fluency**			
Normoglycaemia	··	··	··
Prediabetes	-0·03 (-0·11 to 0·04)	-0·04 (-0·12 to 0·04)	-0·04 (-0·11 to 0·04)
Newly diagnosed diabetes	-0·10 (-0·28 to 0·07)	-0·09 (-0·26 to 0·08)	-0·08 (-0·25 to 0·09)
Known diabetes	-0·17 (-0·31 to -0·03)§	-0·17 (-0·31 to -0·03)¶	-0·15 (-0·29 to -0·01)‖
**Global score**			
Normoglycaemia	··	··	··
Prediabetes	-0·04 (-0·12 to 0·03)	-0·05 (-0·12 to 0·02)	-0·05 (-0·11 to 0·03)
Newly diagnosed diabetes	-0·07 (-0·24 to 0·09)	-0·06 (-0·22 to 0·10)	-0·04 (-0·20 to 0·12)
Known diabetes	-0·18 (-0·31 to -0·06)**	-0·18 (-0·31 to -0·05)††	-0·15 (-0·28 to -0·02)‡‡

Data are beta coefficients (95% CI) based on standardised cognitive scores (mean=0, SD=1). Data for cognitive function are from 1997–99. p values for significant results (p<0·05) compared with reference group are indicated by footnotes. Model 1 is adjusted for age, sex, marital status, and education. Model 2 is adjusted for same parameters as model 1 and health-related behaviours (smoking, alcohol, physical activity, and fruit and vegetable consumption). Model 3 is adjusted for same parameters as model 2 and coronary heart disease, stroke, hypertension, respiratory disease, total cholesterol, obesity, use of antidepressants, and use of lipid-lowering drugs. n for normoglycaemia is 4760; n for prediabetes is 655; n for newly diagnosed diabetes is 116; and n for known diabetes is 192. *p=0·001. †p=0·001. ‡p=0·003. §p=0·015. ¶p=0·018. ‖p=0·032. **p=0·005. ††p=0·007. ‡‡p=0·021.

Table e8: **Estimated difference in cognitive decline over 10 years, as a function of diabetes status in 1997–99 (with multiple imputation for missing data for cognitive tests and covariates)**

	Model 1	Model 2	Model 3
**Memory**			
Normoglycaemia	··	··	··
Prediabetes	0·02 (-0·04 to 0·09)	0·02 (-0·05 to 0·09)	0·02 (-0·05 to 0·09)
Newly diagnosed diabetes	0·04 (-0·11 to 0·19)	0·03 (-0·11 to 0·19)	0·04 (-0·12 to 0·19)
Known diabetes	-0·11 (-0·23 to 0·02)	-0·11 (-0·23 to 0·02)	-0·10 (-0·23 to 0·03)
**Reasoning**			
Normoglycaemia	··	··	··
Prediabetes	-0·02 (-0·06 to 0·03)	-0·02 (-0·06 to 0·02)	-0·02 (-0·06 to 0·02)
Newly diagnosed diabetes	-0·03 (-0·14 to 0·07)	-0·04 (-0·14 to 0·07)	-0·04 (-0·14 to 0·06)
Known diabetes	-0·08 (-0·16 to 0·00)	-0·08 (-0·16 to 0·00)	-0·08 (-0·16 to 0·01)
**Phonemic fluency**			
Normoglycaemia	··	··	··
Prediabetes	0·01 (-0·05 to 0·07)	0·01 (-0·05 to 0·07)	0·01 (-0·04 to 0·07)
Newly diagnosed diabetes	-0·10 (-0·24 to 0·04)	-0·10 (-0·24 to 0·04)	-0·10 (-0·23 to 0·04)
Known diabetes	-0·09 (-0·20 to 0·02)	-0·09 (-0·20 to 0·02)	-0·08 (-0·19 to 0·03)
**Semantic fluency**			
Normoglycaemia	··	··	··
Prediabetes	0·00 (-0·06 to 0·06)	-0·00 (-0·06 to 0·06)	0·00 (-0·06 to 0·06)
Newly diagnosed diabetes	0·03 (-0·10 to 0·16)	0·03 (-0·10 to 0·16)	0·03 (-0·10 to 0·16)
Known diabetes	-0·05 (-0·16 to 0·05)	-0·05 (-0·16 to 0·05)	-0·05 (-0·16 to 0·06)
**Global score**			
Normoglycaemia	··	··	··
Prediabetes	-0·00 (-0·05 to 0·05)	-0·00 (-0·05 to 0·04)	-0·00 (-0·05 to 0·05)
Newly diagnosed diabetes	-0·05 (-0·16 to 0·06)	-0·06 (-0·16 to 0·05)	-0·06 (-0·17 to 0·05)
Known diabetes	-0·11 (-0·19 to -0·02)*	-0·11 (-0·19 to -0·02)†	-0·10 (-0·19 to -0·01)‡

Data are beta coefficients (95% CI) based on standardised cognitive scores (mean=0, SD=1); –0·00 occurs because of rounding. Data for cognitive function are from 1997–99, 2002–04, and 2007–09. p values for significant results (p<0·05) compared with reference group are indicated by footnotes. Model 1 is adjusted for age, sex, marital status, and education. Model 2 is adjusted for same parameters as model 1 and health-related behaviours (smoking, alcohol, physical activity, and fruit and vegetable consumption). Model 3 is adjusted for same parameters as model 2 and coronary heart disease, stroke, hypertension, respiratory disease, total cholesterol, obesity, use of antidepressants, and use of lipid-lowering drugs. n for normoglycaemia is 4760; n for prediabetes is 655; n for newly diagnosed diabetes is 116; and n for known diabetes is 192. *p=0·017. †p=0·017. ‡p=0·024.

Table e9: **Association of glycaemic control (1% increment in HbA_{1c}) with estimated differences in cognitive decline, by diabetes status in 1997–99 (with all covariates entered as time-dependent variables)**

	Model 1	Model 2	Model 3
**Memory**			
Normoglycaemia	-0·02 (-0·08 to 0·04)	-0·02 (-0·08 to 0·04)	-0·00 (-0·07 to 0·06)
Prediabetes	-0·06 (-0·15 to 0·04)	-0·05 (-0·15 to 0·04)	-0·04 (-0·13 to 0·06)
Newly diagnosed diabetes	-0·08 (-0·23 to 0·07)	-0·07 (-0·22 to 0·07)	-0·05 (-0·20 to 0·10)
Known diabetes	-0·13 (-0·23 to -0·02)*	-0·13 (-0·23 to -0·02)†	-0·10 (-0·21 to 0·01)
**Reasoning**			
Normoglycaemia	-0·02 (-0·06 to 0·02)	-0·01 (-0·05 to 0·03)	-0·02 (-0·06 to 0·02)
Prediabetes	-0·01 (-0·07 to 0·06)	-0·00 (-0·07 to 0·06)	-0·01 (-0·07 to 0·06)
Newly diagnosed diabetes	-0·10 (-0·19 to -0·01)‡	-0·10 (-0·19 to -0·01)§	-0·11 (-0·20 to -0·01)¶
Known diabetes	-0·07 (-0·15 to -0·00)	-0·08 (-0·15 to -0·00)‖	-0·07 (-0·14 to -0·00)
**Phonemic fluency**			
Normoglycaemia	-0·02 (-0·08 to 0·03)	-0·02 (-0·07 to 0·03)	-0·02 (-0·07 to 0·03)
Prediabetes	-0·07 (-0·15 to 0·02)	-0·06 (-0·15 to 0·02)	-0·07 (-0·15 to 0·02)
Newly diagnosed diabetes	-0·06 (-0·18 to 0·07)	-0·05 (-0·18 to 0·07)	-0·05 (-0·17 to 0·07)
Known diabetes	0·01 (-0·09 to 0·11)	0·01 (-0·09 to 0·11)	0·01 (-0·08 to 0·11)
**Semantic fluency**			
Normoglycaemia	0·01 (-0·04 to 0·06)	0·02 (-0·04 to 0·07)	0·01 (-0·04 to 0·06)
Prediabetes	0·01 (-0·07 to 0·09)	0·02 (-0·06 to 0·10)	0·01 (-0·07 to 0·09)
Newly diagnosed diabetes	0·03 (-0·10 to 0·15)	0·03 (-0·09 to 0·16)	0·03 (-0·09 to 0·15)
Known diabetes	-0·01 (-0·11 to 0·08)	-0·02 (-0·11 to 0·08)	-0·02 (-0·11 to 0·08)
**Global score**			
Normoglycaemia	-0·01 (-0·05 to 0·03)	-0·01 (-0·05 to 0·04)	-0·00 (-0·05 to 0·04)
Prediabetes	-0·05 (-0·11 to 0·02)	-0·04 (-0·11 to 0·03)	-0·04 (-0·10 to 0·03)
Newly diagnosed diabetes	-0·07 (-0·18 to 0·03)	-0·07 (-0·17 to 0·03)	-0·06 (-0·16 to 0·03)
Known diabetes	-0·06 (-0·14 to 0·02)	-0·06 (-0·14 to 0·02)	-0·06 (-0·13 to 0·02)

Data are beta coefficients (95% CI) based on standardised cognitive scores (mean=0, SD=1); –0·00 occurs because of rounding. Data for cognitive function are from 1997–99, 2002–04, and 2007–09. p values for significant results (p<0·05) compared with reference group are indicated by footnotes. Model 1 is adjusted for age, sex, marital status, and education. Model 2 is adjusted for same parameters as model 1 and health-related behaviours (smoking, alcohol, physical activity, and fruit and vegetable consumption). Model 3 is adjusted for same parameters as model 2 and coronary heart disease, stroke, hypertension, respiratory disease, total cholesterol, obesity, use of antidepressants, and use of lipid-lowering drugs. n for normoglycaemia is 4336; n for prediabetes is 572; n for newly diagnosed diabetes is 100; and n for known diabetes is 152. *p=0·022. †p=0·020. ‡p=0·039. §p=0·038. ¶p=0·027. ‖p=0·040.

Decline in reference group over 10 years, by model

In model 1, decline in the normoglycaemic group over 10 years was –0·29 SD (95% CI –0·32 to –0·26) for memory, –0·34 SD (–0·36 to –0·33) for reasoning, –0·37 SD (–0·39 to –0·34) for phonemic fluency, –0·34 SD (–0·36 to –0·32) for semantic fluency, and –0·45 SD (–0·47 to –0·43) for the global cognitive score (all p<0·0001).

In model 2, decline in the normoglycaemic group over 10 years was –0·29 SD (95% CI –0·32 to –0·26) for memory, –0·34 SD (–0·36 to –0·33) for reasoning, –0·37 SD (–0·39 to –0·35) for phonemic fluency, –0·34 SD (–0·36 to –0·32) for semantic fluency, and –0·45 SD (–0·47 to –0·43) for the global cognitive score (all p<0·0001).

In model 3, decline in the normoglycaemic group over 10 years was –0·29 SD (95% CI –0·32 to –0·26) for memory, –0·34 SD (–0·36 to –0·33) for reasoning, –0·37 SD (–0·39 to –0·35) for phonemic fluency, –0·34 SD (–0·37 to –0·32) for semantic fluency, and –0·45 SD (–0·47 to –0·43) for the global cognitive score (all p<0·0001).
